# Benzodiazepines utilization in Brazilian older adults: a population-based study

**DOI:** 10.11606/s1518-8787.2022056003740

**Published:** 2022-03-11

**Authors:** Marina de Borba Oliveira Freire, Bruna Gonçalves Cordeiro Da Silva, Andréa Dâmaso Bertoldi, Andréia Turmina Fontanella, Sotero Serrate Mengue, Luiz Roberto Ramos, Noemia Urruth Leão Tavares, Tatiane da Silva Dal Pizzol, Paulo Sérgio Dourado Arrais, Mareni Rocha Farias, Vera Lucia Luiza, Maria Auxiliadora Oliveira, Ana Maria Baptista Menezes

**Affiliations:** I Universidade Federal de Pelotas Faculdade de Medicina Programa de Pós-Graduação em Epidemiologia Pelotas RS Brasil Universidade Federal de Pelotas . Faculdade de Medicina . Programa de Pós-Graduação em Epidemiologia . Pelotas , RS , Brasil; II Universidade Federal de Pelotas Faculdade de Medicina Departamento de Medicina Social Pelotas RS Brasil Universidade Federal de Pelotas . Faculdade de Medicina . Departamento de Medicina Social . Pelotas , RS , Brasil; III Universidade Federal do Rio Grande do Sul Faculdade de Medicina Programa de Pós-Graduação em Epidemiologia Porto Alegre RS Brasil Universidade Federal do Rio Grande do Sul . Faculdade de Medicina . Programa de Pós-Graduação em Epidemiologia . Porto Alegre , RS , Brasil; IV Universidade Federal de São Paulo Escola Paulista de Medicina Departamento de Medicina Preventiva São Paulo SP Brasil Universidade Federal de São Paulo . Escola Paulista de Medicina . Departamento de Medicina Preventiva . São Paulo , SP , Brasil; V Universidade de Brasília Faculdade de Ciências da Saúde Departamento de Farmácia Brasília DF Brasil Universidade de Brasília . Faculdade de Ciências da Saúde . Departamento de Farmácia . Brasília , DF , Brasil; VI Universidade Federal do Rio Grande do Sul Faculdade de Farmácia Departamento de Produção e Controle de Medicamentos Porto Alegre RS Brasil Universidade Federal do Rio Grande do Sul . Faculdade de Farmácia . Departamento de Produção e Controle de Medicamentos . Porto Alegre , RS , Brasil; VII Universidade Federal do Ceará Faculdade de Farmácia, Odontologia e Enfermagem Departamento de Farmácia Fortaleza CE Brasil Universidade Federal do Ceará . Faculdade de Farmácia, Odontologia e Enfermagem . Departamento de Farmácia . Fortaleza , CE , Brasil; VIII Universidade Federal de Santa Catarina Centro de Ciências da Saúde Departamento de Ciências Farmacêuticas Florianópolis SC Brasil Universidade Federal de Santa Catarina . Centro de Ciências da Saúde . Departamento de Ciências Farmacêuticas . Florianópolis , SC , Brasil; IX Fundação Oswaldo Cruz Escola Nacional de Saúde Pública Sergio Arouca Departamento de Política de Medicamentos e Assistência Farmacêutica Rio de Janeiro RJ Brasil Fundação Oswaldo Cruz . Escola Nacional de Saúde Pública Sergio Arouca . Departamento de Política de Medicamentos e Assistência Farmacêutica . Rio de Janeiro , RJ , Brasil

**Keywords:** Aged, Drug Utilization, Benzodiazepines, Risk Factors, Cross-Sectional Studies

## Abstract

**OBJECTIVE:**

To evaluate the utilization of benzodiazepines (BZD) in Brazilian older adults, based on the *Pesquisa Nacional de Acesso, Utilização e Promoção do Uso Racional de Medicamentos* (PNAUM - National Survey of Access, Use and Promotion of Rational Use of Medicines).

**METHODS:**

The PNAUM is a cross-sectional study conducted between 2013 and 2014, representing the Brazilian urban population. In the present study, we included 60 years or older (n = 9,019) individuals. We calculated the prevalence of BZD utilization in the 15 days prior to survey data collection according to independent variables, using a hierarchical Poisson regression model. A semistructured interview performed empirical data collection (household interview).

**RESULTS:**

The prevalence of BZD utilization in the older adults was 9.3% (95%CI: 8.3–10.4). After adjustments, BZD utilization was associated with female sex (PR = 1.88; 95%CI: 1.52–2.32), depression (PR = 5.31; 95%CI: 4.41–6, 38), multimorbidity (PR = 1.44; 95%CI: 1.20–1.73), emergency room visit or hospitalization in the last 12 months (PR = 1.42; 95%CI: 1.18–1.70 ), polypharmacy (PR = 1.26; 95%CI: 1.01–1.57) and poor or very poor self-rated health (PR = 4.16; 95%CI: 2.10–8.22). Utilization was lower in the North region (PR = 0.18; 95%CI: 0.13–0.27) and in individuals who reported abusive alcohol consumption in the last month (PR = 0.42; 95%CI: 0.19–0.94).

**CONCLUSION:**

Despite contraindications, results showed a high prevalence of BZD utilization in older adults, particularly in those with depression, and wide regional and sex differences.

## INTRODUCTION

The first benzodiazepines (BZDs) were synthesized in the 1950s and ^1^ achieved great popularity in the following years due to their proven efficacy in the treatment of anxiety, insomnia, aggression and seizures ^2^ , in addition to adjuvant utilization in other clinical conditions, such as muscle relaxation and analgesia ^3^ . The low incidence of respiratory depression with BZDs taken orally provided a sense of security and contributed to making the class one of the most prescribed around the world ^2^ .

However, prolonged utilization was associated with a wide range of adverse events and weak evidence of long-term benefit ^4^ . Adverse events documented on BZDs utilization include dementia, cognitive decline, psychomotor disorders, daytime sleepiness, car accidents, tolerance, and dependence ^3^ , besides a higher incidence of fractures and falls, mobility restriction, and reduced social participation in the older adults ^5^ . Therefore, BZDs are classified as potentially inappropriate drugs, whose prescription should be avoided in older adults ^6^ . However, despite this recommendation, the literature points out an increase in BZDs utilization along ageing ^7^ , despite the higher risk of adverse events associated with changes in pharmacodynamics and pharmacokinetics induced by age and polypharmacy ^8^ .

In this scenario, the *Comitê Nacional para a Promoção do Uso Racional de Medicamentos* (National Committee for the Promotion of the Rational Use of Medicines), on behalf of the Ministry of Health, launched the “ *Uso de Medicamentos e Medicalização da Vida: Recomendações e Estratégias* ” (“Drug utilization and Medicalization of Life: Recommendations and Strategies”) in 2019, prompting the rational utilization of medicines by encouraging education, information, regulation, and research. The publication highlighted the need to restrict the prescription of BZD to individuals aged 60 years and over ^9^ .

So far, no studies with national representation evaluated BZD utilization in older adults. The present study aims to fill this gap in the literature, evaluating the prevalence of BZD utilization in Brazilian older adults and its distribution according to sociodemographic, behavioral, and health-related variables based on data from the *Pesquisa Nacional de Acesso, Utilização e Promoção do Uso Racional de Medicamentos* (PNAUM - National Research on Access, Use and Promotion of Rational Use of Medicines).

## METHODS

The PNAUM was a cross-sectional, population-based study conducted between September 2013 and February 2014, which included individuals of all ages, living in urban areas of the Brazilian territory (n = 41,433). The sampling process was carried out in three stages (municipalities, census tracts and households), followed by a post-stratification process by region, sex, and age, ensuring the representativeness of the Brazilian population. Data collection was carried out through a face-to-face household interview, with data recording on a tablet using specific software for the survey questionnaires. Information on respondents unable to communicate was obtained by a surrogate informant. Additional information is available elsewhere ^10^ .

In the present study, we analyzed data referring to older adults (60 years and over) interviewed in the survey (n = 9,019).

The dependent variable analyzed was the BZD utilization in the 15 days prior to the collection of the research data, constructed with information from two investigated variables: 1) chronic medication utilization, in which all medications referred to by the interviewee as being in current use were evaluated for the treatment of a certain chronic disease; and 2) medications for occasional use, based on the following question: “In the last 15 days, have you taken any medication for sleeping or for nerves?”. When the answer was positive, an additional question was asked: “How long will the treatment last?” (taken only once; until improvement/cure; will not take it anymore; whenever symptoms return; forever).

In order to ensure a better quality of information, the interviewee was asked to show the box(es) or prescription(s) of the medication(s).

BZDs were considered to be a medication classified according to the *Anatomical Therapeutic Chemical Index* (ATC Index), developed by the *World Health Organization Collaboration Center for Drugs* ^11^ Statistic Methodology, under the codes N05BA (anxiolytic BZD derivatives) and N05CD (hypnotic BZD derivatives) in Brazil, in addition to clonazepam, classified as an anticonvulsant under code N03AE01. Information was also collected on the use of medications related to BZDs marketed in Brazil (zolpidem and zopiclone), under code N05CF ^11^ , although these drugs were not included in the main outcome because they do not belong to the BZD class.

The independent variables encompassed sociodemographic, behavioral and health-related factors:

a.Sociodemographic: region (North; Northeast; Midwest; Southeast; South); sex (male; female); age (years: 60 to 69; 70 to 79; 80 or older); self-reported skin color (white; black; mixed; yellow; Indigenous); marital status (without a partner; with a partner); schooling (years of study: 0 to 4; 5 to 8; 9 to 11; 12 or more); socioeconomic level (AB [richest]; C; D; E [poorest]), according to the classification of the *Associação Brasileira de Empresas de Pesquisa* (ABEP - Brazilian Association of Research Companies) ^12^ ;

b.Behavioral: smoking (current smoker; former smoker; non-smoker); abusive consumption of alcohol in the last month (yes; no), defined as abusive consumption of four or more doses of alcoholic beverage (woman) or five or more doses (man), on a single occasion, at least once in the last month. By “dose”, we understand an alcoholic beverage equivalent to a can of beer, a glass of wine or a dose of liquor ( *cachaça* ), whiskey or any other distilled alcoholic beverage;

c.Health-related: health insurance (yes; no); depression (yes; no), assessed through the question: “Has a doctor ever told you that you have depression?”; multimorbidity (yes; no), considering the presence of two or more chronic diseases as multimorbidity. Diseases included: high blood pressure, diabetes, heart disease, high cholesterol, ischemia or stroke, asthma, chronic obstructive pulmonary disease (emphysema or chronic bronchitis), and rheumatologic disease. The presence or absence of these diseases was assessed through the question: “Has a doctor ever told you that you have [disease]?”; emergency room visit or hospital admission in the last 12 months (yes; no); polypharmacy (yes; no), considering as polypharmacy the current use of five or more drugs (excluding BZDs), including homeopathy, formulas made in a compounding pharmacy, florals, vitamins, and herbal medicines; and self-rated health (very good; good; fair; bad/very bad).

Descriptive analysis of the independent variables was performed to characterize the sample; proportions and respective 95% confidence intervals were also calculated. The prevalence of BZD utilization in the 15 days prior to data collection was calculated, with its respective 95% confidence interval, general, and according to the independent variables. With the use of post-stratification weights, the direct relationship between the number of observations and the percentages is lost. Thus, the number of sample observations (n) was kept only in the titles of the tables. Poisson regression with robust variance was used to obtain the raw and adjusted prevalence ratios. In the adjusted analysis, a five-level hierarchical model was built based on the literature. The first level included the variables region of the country, sex, age, and skin color. The second level included the variables socioeconomic level (ABEP), schooling, and marital status. The third level integrated the variables health insurance, smoking, depression, and multimorbidity. In the fourth level, the variable emergency room visit or hospital admission in the last 12 months. The fifth level encompasses the variables polypharmacy, abusive consumption of alcohol in the last month, and self-rated health. The variables were adjusted for those of the same level and of the higher level(s). We used backward selection and kept the variables whose p were less than 0.20 in the final model. We considered a significance level of 5%. Data were analyzed using Stata version 15.1 (StataCorp, College Station, Texas, USA).

The PNAUM was approved by the National Research Ethics Committee (Protocol 18947013.6.0000.0008) and by the Research Ethics Committee of the Universidade Federal do Rio Grande do Sul (Protocol 19997). All interviews were carried out after the interviewee or his legal guardian had read and signed an informed consent form.

## RESULTS

A total of 9,019 individuals were included in this study. After adjustments in region, sex, and age, they represented approximately the 23 million older adults living in the urban area of Brazil.

Women (57.7%), individuals aged between 60 and 69 years (52.3%), of white skin color (52.8%), belonging to the C socioeconomic level (54.7%), who lived with a partner (55.8%) and resided in the Southeast region (52.5%) predominated. Of the total, 9.5% and 41.5% had depression and multimorbidity respectively; 21.8% were polypharmacy users. About 4% reported abusive consumption of alcohol in the last month, with a wide difference between the sexes (women: 1%; 95%CI 0.7–1.5 versus men: 8.3%; 95%CI 7.0–9,8). Almost half of the sample had a self-rated health of good health ( Table 1 ).

**Table 1:** Description of the sample, according to sociodemographic, behavioral, and health-related variables. PNAUM, 2014 (n = 9,019).

Variables	% ^a^	95%CI
Region		
North	4.8	3.8–6.1
Northeast	21.2	17.2–25.8
Midwest	6.8	5.4–8.5
Southeast	52.5	46.5–58.4
South	14.7	11.8–18.1
Sex		
Male	42.2	40.7–43.8
Female	57.7	56.2–59.3
Age		
60–69 years	52.3	50.7–53.9
70–79 years	32.4	31.0–33.9
80 years or more	15.2	14.1–16.4
Skin color		
White	52.8	49.8–55.8
Black	9.7	8.5–11.0
Brown	36.0	33.4–38.7
Yellow	1.2	0.9–1.6
Indigenous	0.3	0.2–0.4
Socioeconomic level (ABEP)		
A–B	21.6	19.5–23.9
C	54.7	52.7–56.6
D	19.0	17.1–21.1
E	4.6	4.0–5.5
Schooling		
0–4 years	38.9	36.8–40.9
5–8 years	20.1	18.8–21.4
9–11 years	30.1	28.5–31.7
12 years or more	10.9	9.7–12.2
Marital Status		
Without partner	44.2	42.3–46.1
With partner	55.8	53.9–57.6
Health insurance		
No	73.0	70.1–75.6
Yes	27.0	24.3–29.9
Smoking		
Never smoked	62.5	60.3–64.7
Former smoker	26.9	25.1–28.8
Current smoker	10.6	9.6–11.6
Depression		
No	90.5	89.4–91.4
Yes	9.5	8.6–10.6
Multimorbidity		
No	58.5	56.5–60.5
Yes	41.5	39.5–43.5
Emergency room visit or hospital admission in the last 12 months		
No	78.7	77.2–80.2
Yes	21.3	19.8–22.8
Polypharmacy		
No	78.2	76.5–79.8
Yes	21.8	20.2–23.5
Abusive alcohol consumption in the last month		
No	95.9	95.2–96.5
Yes	4.1	3.5–4.8
Self-rated health		
Very good	8.0	7.2–8.9
Good	48.9	47.0–50.7
Average	36.0	34.4–37.5
Poor, very poor	7.1	6.4–7.9

^a^ Percentages adjusted by sample weights and by post-stratification, according to age and sex.

The prevalence of BZD utilization in older adults prior to 15 days of the survey data collection was 9.3% (95%CI 8.3–10.4). Considering the total number of BZD users, 59.3% (95%CI 54.0–64.4%) reported occasional utilization. Even so, 36.8% (95%CI 30.8–43.1) of them responded that the treatment would last “forever” and 27.2% (95%CI 21.9–33.2) that they would take the medication “whenever the symptoms returned”. Only 0.7% (95%CI 0.2–2.4) reported that they would no longer take the medication.

**Figure 1:**
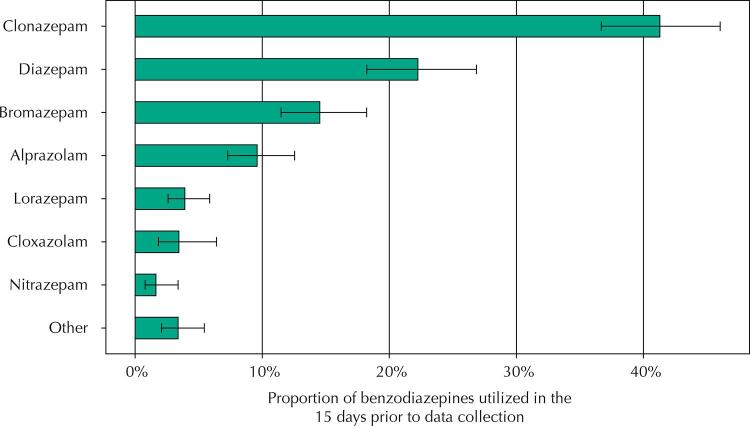
The proportion of benzodiazepines utilization by Brazilian older adults in the 15 days prior to data collection. PNAUM, 2014 (n = 699).

Considering the total amount of BZD, those with the highest proportion of utilization were clonazepam (41.3%; 95%CI 36.7–46.0), diazepam (22.2%; 95%CI 18.2–26.9), bromazepam (14.5%; 95%CI 11.5–18.2), and alprazolam (9.6%; 95%CI 7.3–12.5) ( Figure 1 ). This pattern remained similar in all regions of the country. Regarding the BZD-related drugs, the prevalence of zolpidem utilization in older adults was 0.1% (95%CI 0.06–0.2).

**Figure 2:**
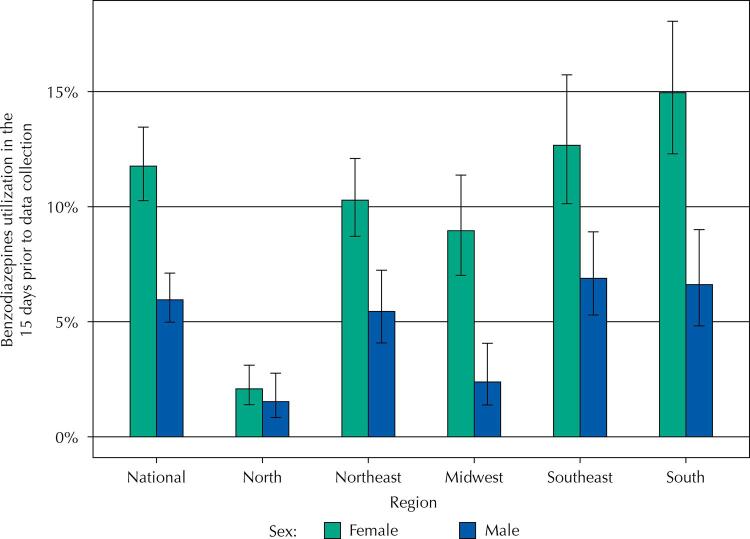
Benzodiazepines utilization in Brazilian older adults in the 15 days prior to data collection, according to region and sex. PNAUM, 2014 (n = 9,019).

Figure 2 shows the prevalence of BZD utilization in older adults by region, with sex stratification. We point that the prevalence of BZD utilization is consistently higher in women, although with important regional differences: while in the North Region the difference between the sexes is small, not reaching statistical significance, in the South and Central-West regions the variation is wide, reaching prevalences approximately two and three times higher in women than in men, respectively.

The highest prevalence of BZD utilization was found in the following categories: female, South and Southeast regions, 70 years of age or older, white skin color, living without a partner, having health insurance, having depression or multimorbidity, visiting the emergency room or hospitalization in the last 12 months, polypharmacy, not reporting abusive consumption of alcohol in the last month and poor or very poor self-rated health ( Table 2 ).

**Table 2:** Prevalence and crude prevalence ratios for the use of benzodiazepines in Brazilian older adults for the 15 days prior to survey data collection, according to sociodemographic, behavioral, and health-related variables. PNAUM, 2014 (n = 9,019).

Variable	Prevalence (%) ^a^	CI95%	Gross PR	CI95%
Region				p < 0.001 ^b^
North	1.8	1.3–2.6	0.18	0.12–0.26
Northeast	8.2	7.1–9.7	0.81	0.66–0.99
Midwest	6.1	4.8–7.6	0.60	0.46–0.77
Southeast	10.2	8.5–12.2	1.00	
South	11.5	9.6–13.7	1.13	0.92–1.38
Sex				p < 0.001 ^b^
Male	6.0	5.0–7.1	1.00	
Female	11.8	10.3–13.4	1.97	1.60–2.43
Age				p = 0.003 ^b^
60–69 years	7.9	6.8–9.2	1.00	
70–79 years	11.0	9.3–12.9	1.39	1.13–1.71
80 years or more	10.6	8.3–13.3	1.34	1.04–1.73
Skin color				p = 0.004 ^b^
White	11.1	9.8–12.4	1.00	
Black	7.8	5.4–11.2	0.71	0.50–1.02
Brown	8.0	6.7–9.5	0.72	0.59–0.89
Socioeconomic level (ABEP)				p = 0.867 ^b^
A–B	8.6	7.2–9.3	1.00	
C	9.5	8.4–10.8	1.11	0.87–1.40
D	9.4	7.4–12.0	1.09	0.82–1.44
E	8.8	5.7–13.4	1.02	0.62–1.67
Schooling				p = 0.185 ^b^
0–4 years	10.4	8.8–9.1	1.00	
5–8 years	8.2	6.7–10.2	0.79	0.62–1.01
9–11 years	8.7	6.9–10.9	0.84	0.66–1.07
12 years or more	8.4	6.3–10.9	0.80	0.59–1.10
Marital Status				p < 0.001 ^b^
Without partner	11.5	9.8–13.4	1.00	
With partner	7.9	6.9–9.1	0.69	0.57–0.83
Health Insurance				p = 0.005 ^b^
No	8.5	7.5–9.6	1.00	
Yes	11.5	9.4–13.9	1.34	1.09–1.64
Smoking				p = 0.450 ^a^
Never smoked	9.3	8.0–10.8	1.00	
Former smoker	8.1	6.4–10.1	0.87	0.68–1.11
Current smoker	9.8	7.6–12.7	1.05	0.79–1.41
Depression				p < 0.001 ^b^
No	6.0	5.3–10.0	1.00	
Yes	39.9	34.7–45.4	6.59	5.57–7.80
Multimorbidity				p < 0.001 ^b^
No	6.2	5.3–7.3	1.00	
Yes	13.6	11.9–15.6	2.88	2.37–3.50
Emergency room visit or hospital admission in the last 12 months				p < 0.001 ^b^
No	7.7	6.8–8.8	1.00	
Yes	15.1	12.8–17.8	1.95	1.60–2.38
Polypharmacy				p < 0.001 ^b^
No	6.8	6.0–7.8	1.00	
Yes	18.2	15.4–21.3	2.65	2.21–3.19
Abusive alcohol consumption in the last month				p < 0.001 ^b^
No	9.4	8.3–10.7	1.00	
Yes	2.3	1.0–5.1	0.25	0.11–0.55
Self-rated health				p < 0.001 ^c^
Very good	2.3	1.2–4.2	1.00	
Good	6.8	5.6–8.2	2.98	1.59–5.66
Average	11.5	10.0–13.2	5.03	2.67–9.49
Poor, very poor	23.0	17.9–29.0	10.0	5.16–19.50

^a^ Percentages adjusted by sample weights and by post-stratification, according to age and sex. Only variables with p < 0.20 were kept in the adjusted model.

^b^ p value for the χ ^2^ test of heterogeneity

^c^ p value for trend.

^d^ Considering the reduced n, the “yellow” and “Indigenous” categories were transformed into *missing* for the regression model.

**Figure 3:**
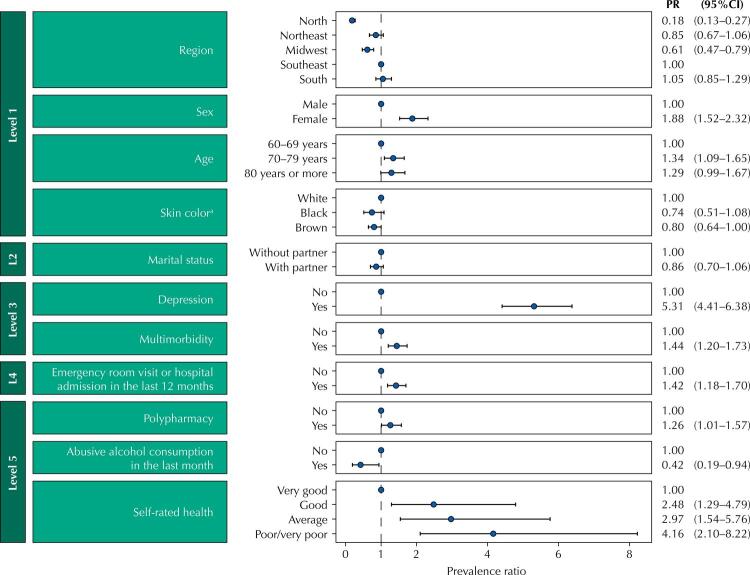
Adjusted prevalence ratios for benzodiazepines utilization in Brazilian older adults in the 15 days prior to data collection. PNAUM, 2014 (n = 9,019).

^a^ Considering the reduced n, the “yellow” and “Indigenous” categories were transformed into missing for the regression model.

After adjustments, the variables that did not remain in the hierarchical model were socioeconomic level, schooling, smoking, and health insurance (p > 0.20). Figure 3 shows the adjusted prevalence ratios of the variables kept in the hierarchical model. Among them, only skin color and marital status were not significantly associated with BZD utilization in the adjusted analysis. The BZD utilization was five times higher in individuals with depression. It increased with the worsening of self-rated health. Individuals who reported abusive consumption of alcohol showed a 58% reduction in the risk of using BZD.

## DISCUSSION

This is the first population-based study representing the five Brazilian regions that evaluated the prevalence of BZD utilization in older adults. The prevalence found was 9.3%, higher than found in Saudi Arabia (4%) ^13^ and lower than that reported in France (31%) ^14^ , Finland (31%) ^15^ and Taiwan (43%) ^16^ . However, in absolute terms, it corresponds to more than two million older adults in Brazil, representing a large impact in terms of public health.

The most frequently utilized medications were clonazepam and diazepam, medications among the BZDs that are provided free of charge by the Unified Health System (SUS) ^17^ . Most Brazilian studies found a similar pattern ^18^ . Only one study found higher consumption of alprazolam and bromazepam ^2^ , pattern also observed in most high-income countries where there is a tendency to opt for newer medications, with a short half-life. However, this study only used data from private pharmacies ^2^ .

On the other hand, women, individuals aged between 70 and 79 years, with depression or multimorbidity, who reported at least one emergency visit or hospitalization in the last 12 months, polypharmacy and poor or very poor self-rated health, had a lower utilization prevalence than residents in the North Region and individuals who reported abusive consumption of alcohol in the last month.

The present study has some disadvantages, related to the growing increase in losses and refusals observed in population surveys ^10^ . Published sample characterization data show household response rates of 51.7% and 51.5% for men and women, respectively. This is an underestimated proportion because it included vacant households in the calculation. A weak but statistically significant negative correlation was also found between the average income of the census tract and the response rate in all regions of the country ^10^ . However, other studies that evaluated older adults did not find a statistically significant association between socioeconomic status and BZD utilization ^3^ , reducing the possibility of selection bias. Residents’ response rates were similar between men and women (90.1% and 93.7%, respectively) ^10^ .

Regarding the interviewee’s sex, women were about twice as likely as men to utilize BZD, a consistent finding in the literature ^19^ . Some questions are guided to explain this difference. Firstly, studies show that women have more depressive and anxiety disorders, while men have more addictive and externalizing disorders ^3^ . Since BZDs are used especially in the treatment of the former, their utilization may be a *proxy* for sex differences in the prevalence of mental disorders ^20^ , strongly impacted by the degree of sex inequality within a patriarchal society. Another relevant issue is that women seek more health services to treat mental health problems ^3 , 20^ . In the context of older adults, this finding has particular clinical relevance, considering the increased risk of falls after BZD utilization and the high risk of fractures in older women, especially hip fractures, which occur with high morbidity and mortality ^21^ .

Broad regional differences were detected, with the prevalence of BZD utilization in the North Region (1.8%) being much lower than that found in other regions. Studies carried out in Brazil that evaluated the BZD utilization in the general population found a similar pattern ^2 , 3^ . They pointed out the increased consumption in cities with greater population density and a higher percentage of physicians ^2^ . Factors such as chaotic traffic, a feeling of insecurity, a competitive environment, great consumerist appeal, and low social cohesion make up the lifestyle of today’s large cities. These factors compromise the well-being of inhabitants and may contribute to the greater BZD utilization observed in these places ^2^ . It is also important to consider that the sale of BZDs has been controlled in Brazil since 1998 ^1^ , so access to medical prescriptions is a determining factor in their utilization. Medication access is likely to be difficult in regions that have a lower number of physicians per inhabitant: while the country has an average ratio of 2.27 physicians per thousand inhabitants, the North Region has a rate of 1.30, 43% lower than the national average ratio ^22^ .

Previous diagnosis of depression was the most important independent variable to predict the BZD utilization in older adults. Individuals with a history of depression had a prevalence of use of 39.9% and, even after an adjusted analysis, were five times more likely to use BZD than individuals without a diagnosis of depression. Similar findings were found in other studies that evaluated the BZD utilization in the general population and in the older adults ^23^ .

Antidepressant drugs (AD) are the treatment of choice for depression and are also effective for the treatment of coexisting anxiety symptoms. However, the beneficial effects of AD are usually only obtained after several weeks, during which time BZDs are usually prescribed for a immediate relief of symptoms ^24^ . However, the benefit of the combined treatment is not sustained for more than four weeks ^25^ . Nevertheless, the prevalence of long-term BZD utilization in older adults is high ^24^ . Qualitative studies pointed out the renewal of prescriptions initiated by other physicians as a possible cause, seeking to meet patients’ requests and avoid damage to the doctor-patient relationship ^26^ . It is important to emphasize that dependence on BZDs occurs within a few weeks of regular use and is associated with withdrawal syndrome, characterized by the occurrence of sleep disorders, anxiety, and agoraphobia after medication discontinuation, especially when performed abruptly. The intensity of symptoms can explain relapses after attempts to stop, resulting in the perpetuation of use ^27^ .

Multimorbidity and polypharmacy showed an increase of 1.4 and 1.3 times, respectively, in the probability of using BZD. This finding reflects a greater burden of disease and greater risk of anxiety disorders ^19^ . Both conditions are more prevalent in older adults and are associated with a higher risk of iatrogenesis, functional deterioration, loss of independence, and autonomy ^8^ . Users of a greater number of medications are still at high risk of medical interactions and adverse events such as confusion, agitation, and delirium. These symptoms can be confused with anxiety and lead to the prescription of BZD, worsening the conditions ^19^ .

Individuals who reported abusive consumption of alcohol in the last month showed a reduction in the probability of using BZD (PR = 0.42; 95%CI 0.19–0.94). Most studies that evaluated alcohol consumption in the last month found similar findings ^14 , 18 , 19^ . It is known that alcohol and BZDs share common mechanisms of action ^3^ and potentially produce comparable effects on baseline anxiety and sleep, leading individuals to feel the need for one or the other, but not for both ^19^ . In part, this choice seems to be influenced by the individual’s sex. The present study found a higher prevalence of BZD utilization in women and alcohol consumption by men. It is also possible that this difference results from a lower number of BZD prescriptions among alcohol users, since the effect of the two substances as respiratory center depressants is synergistic, resulting in serious and potentially fatal intoxications ^18^ .

Only one study found the opposite association, in which alcohol abuse determined greater BZD utilization (OR 3.1–95%CI 1.7–5.7) ^3^ . However, this study included the general population and used a 12-month recall period for both alcohol abuse and BZD utilization. In a mediation analysis, the authors noted that the effect of alcohol on BZD utilization was both direct and indirect, including depressive symptoms, physical inactivity, and sleep disturbances. Because they have common mechanisms of action, we point out that alcohol and BZD could lead to cross-tolerance. Thus, BZD-dependent individuals may choose to use alcohol during periods of deprivation, in the same way that the BZD class is part of the treatment for alcohol withdrawal syndrome ^3^ . This phenomenon could also justify our findings, given alcohol abuse is the most proximal determinant in the hierarchical model. Thus, an integrated approach to the treatment of both conditions is needed.

Emergency room visits or hospital admissions in the last 12 months were also associated with a higher BZD prescription for older adults, with a 1.4-fold increased risk. Recent studies have shown a high prevalence of older adults receiving BZD during hospitalization. This is a serious issue because new BZD prescriptions given to older patients at hospital discharge can lead to chronic medication utilization ^28^ .

Therefore, it is important to emphasize that the prevalence of over-the-counter BZD utilization found in the literature varies from 3.3% to 8.4% ^2^ , demonstrating that efforts aimed at increasing supervision over the sale of the drug are not enough to solve the problem, since most users obtain that medication by medical prescription. Thus, the rational BZD utilization starts from the appreciation of continuing medical education and the encouragement of multi-professional partnerships ^2 , 9^ , based on comprehensive care for older patients, with the objective of reducing avoidable adverse events, and maximizing the independence and autonomy of these individuals ^8^ .

The relevance of the topic requires further investigation, especially in the covid-19 post-pandemic context ^29^ . Emerging evidence have shown dramatic impacts on individuals’ mental health, with increased anxiety and social isolation due to physical distancing policies introduced for infection control ^30^ , particularly affecting the older adults. It is likely that the high prevalence of BZD utilization will increase even more in this context. Once the public health emergency related to the pandemic itself is controlled, public policies promoting the rational utilization of this drug class are needed.

Despite the potential limitations inherent to a cross-sectional study, especially in relation to causal inference, the present study brought consistent and nationally representative data, hitherto unexplored by literature. Despite recommendations against its use ^6^ , the results revealed a high prevalence of BZD utilization in older individuals, particularly in those with depression, in addition to wide differences in relation to the region of the country and the individual’s sex.
