# Supplementary material for: Benzodiazepines utilization in Brazilian older adults: a population-based study
Source: Rev Saude Publica. 2022 Mar 11;56:10. doi: 10.11606/s1518-8787.2022056003740 (PMC8926397; doi:10.11606/s1518-8787.2022056003740)
Supplement: Translation

# Translation: Utilização de benzodiazepínicos em idosos brasileiros: um estudo de base populacional

**Authors:** Marina de Borba Oliveira Freire, Bruna Gonçalves Cordeiro Da Silva, Andréa Dâmaso Bertoldi, Andréia Turmina Fontanella, Sotero Serrate Mengue, Luiz Roberto Ramos, Noemia Urruth Leão Tavares, Tatiane da Silva Dal Pizzol, Paulo Sérgio Dourado Arrais, Mareni Rocha Farias, Vera Lucia Luiza, Maria Auxiliadora Oliveira, Ana Maria Baptista Menezes

**Affiliations:** I Brasil Universidade Federal de Pelotas. Faculdade de Medicina. Programa de Pós-Graduação em Epidemiologia. Pelotas, RS, Brasil; II Brasil Universidade Federal de Pelotas. Faculdade de Medicina. Departamento de Medicina Social. Pelotas, RS, Brasil; III Brasil Universidade Federal do Rio Grande do Sul. Faculdade de Medicina. Programa de Pós-Graduação em Epidemiologia. Porto Alegre, RS, Brasil; IV Brasil Universidade Federal de São Paulo. Escola Paulista de Medicina. Departamento de Medicina Preventiva. São Paulo, SP, Brasil; V Brasil Universidade de Brasília. Faculdade de Ciências da Saúde. Departamento de Farmácia. Brasília, DF, Brasil; VI Brasil Universidade Federal do Rio Grande do Sul. Faculdade de Farmácia. Departamento de Produção e Controle de Medicamentos. Porto Alegre, RS, Brasil; VII Brasil Universidade Federal do Ceará. Faculdade de Farmácia, Odontologia e Enfermagem. Departamento de Farmácia. Fortaleza, CE, Brasil; VIII Brasil Universidade Federal de Santa Catarina. Centro de Ciências da Saúde. Departamento de Ciências Farmacêuticas. Florianópolis, SC, Brasil; I Brasil Fundação Oswaldo Cruz. Escola Nacional de Saúde Pública Sergio Arouca. Departamento de Política de Medicamentos e Assistência Farmacêutica. Rio de Janeiro, RJ, Brasil

**Keywords:** Idoso, Uso de Medicamentos, Benzodiazepinas, Fatores de Risco, Estudos Transversais

## Abstract

**OBJETIVO:**

Avaliar a utilização de benzodiazepínicos (BZD) em idosos brasileiros,a partir de dados da Pesquisa Nacional de Acesso, Utilização e Promoção do Uso Racional de Medicamentos (PNAUM).

**MÉTODOS:**

A PNAUM é um estudo transversal, conduzido entre 2013 e 2014, com representatividade da população urbana brasileira. No presente estudo, foram incluídos indivíduos com 60 anos ou mais (n = 9.019). Foi calculada a prevalência de utilização de BZD nos 15 dias anteriores à coleta dos dados da pesquisa, geral e segundo as variáveis independentes, por meio de análise bruta e ajustada, utilizando modelo hierárquico de regressão de Poisson. A coleta de dados foi realizada por meio de entrevista domiciliar.

**RESULTADOS:**

A prevalência de utilização de BZD em idosos foi de 9,3% (IC95%: 8,3–10,4). Após análise ajustada, foram associados à maior utilização de BZD: sexo feminino (RP = 1,88; IC95%: 1,52–2,32), depressão (RP = 5,31; IC95%: 4,41–6,38), multimorbidade (RP = 1,44; IC95%: 1,20–1,73), visita à emergência ou internação hospitalar nos últimos 12 meses (RP = 1,42; IC95%: 1,18–1,70), polifarmácia (RP = 1,26; IC95%: 1,01–1,57) e autopercepção de saúde ruim ou muito ruim (RP = 4,16; IC95%: 2,10–8,22). A utilização foi menor na região Norte (RP = 0,18; IC95%: 0,13–0,27) e em indivíduos que relataram consumo abusivo de álcool no último mês (RP = 0,42; IC95%: 0,19–0,94).

**CONCLUSÃO:**

Apesar das recomendações contrárias ao uso, os resultados demonstraram elevada prevalência de utilização de BZD em idosos, particularmente naqueles que apresentam depressão, além de amplas diferenças em relação às regiões do país e ao sexo do indivíduo.

## INTRODUÇÃO

Os primeiros benzodiazepínicos (BZD) foram sintetizados na década de 1950 ^1^ e alcançaram grande popularidade nos anos seguintes devido a sua comprovada eficácia no tratamento da ansiedade, insônia, agressividade e convulsões ^2^ , além de uso adjuvante em outras condições clínicas, como relaxamento muscular e analgesia ^3^ . A baixa incidência de depressão respiratória com BZD ingeridos por via oral propiciou uma sensação de segurança e contribuiu para tornar a classe uma das mais prescritas ao redor do mundo ^2^ .

Entretanto, o uso prolongado mostrou-se associado a extensa gama de eventos adversos, aliado à fraca evidência de benefício em longo prazo ^4^ . Os eventos adversos documentados com a utilização de BZD incluem demência, declínio cognitivo, transtornos psicomotores, sonolência diurna, acidentes de carro, tolerância e dependência ^3^ , além de maior incidência de fraturas e quedas, restrição da mobilidade e redução da participação social em idosos ^5^ . Por isso, os BZD são classificados como medicamentos potencialmente inapropriados, cuja prescrição deve ser evitada em idosos ^6^ , porém, apesar dessa recomendação, a literatura aponta o aumento da utilização de BZD com o aumento da idade ^7^ , dado preocupante considerando o maior risco de eventos adversos associado às modificações da farmacodinâmica e farmacocinética induzidas pela idade e polifarmácia ^8^ .

Nesse cenário, o Ministério da Saúde, por meio do Comitê Nacional para a Promoção do Uso Racional de Medicamentos, lançou em 2019 a publicação intitulada “Uso de Medicamentos e Medicalização da Vida: Recomendações e Estratégias”, com objetivo de promover o uso racional de medicamentos por meio do estímulo à educação, informação, regulação e pesquisa; a publicação destacou a necessidade de restringir a prescrição de BZD para indivíduos com 60 anos ou mais ^9^ .

Até o momento, não foram encontrados estudos com representatividade nacional que tenham avaliado a utilização de BZD em idosos. O presente estudo tem como objetivo preencher essa lacuna na literatura, avaliando a prevalência de utilização de BZD em idosos brasileiros e sua distribuição, segundo variáveis sociodemográficas, comportamentais e relacionadas à saúde, a partir de dados da Pesquisa Nacional de Acesso, Utilização e Promoção do Uso Racional de Medicamentos (PNAUM).

## MÉTODOS

A PNAUM foi um estudo transversal, de base populacional, conduzido entre setembro de 2013 e fevereiro de 2014, que incluiu indivíduos de todas as idades, residentes na zona urbana do território brasileiro (n = 41.433). O processo de amostragem foi realizado em três estágios (municípios, setores censitários e domicílios), seguido por processo de pós-estratificação por região, sexo e idade, garantindo a representatividade da população brasileira. A coleta de dados foi realizada por meio de entrevista domiciliar, face a face, com o registro dos dados em tablet com software específico para os questionários da pesquisa. As informações dos entrevistados incapazes de se comunicar foram obtidas por meio de informante substituto. Informações adicionais encontram-se disponíveis em publicação anterior ^10^ .

No presente estudo, foram analisados os dados referentes à população idosa (60 anos ou mais) entrevistada durante o inquérito (n = 9.019).

A variável dependente analisada foi a utilização de BZD nos 15 dias anteriores à coleta dos dados da pesquisa, construída com informações provenientes de duas variáveis investigadas: 1) medicamentos de uso crônico, em que foram avaliados todos os medicamentos referidos pelo entrevistado como de uso corrente para o tratamento de determinada doença crônica; e 2) medicamentos de uso eventual, a partir da seguinte pergunta: “Nos últimos 15 dias, o(a) Sr.(a) tomou algum remédio para dormir ou para os nervos?”. Quando a resposta era positiva, foi feita uma pergunta adicional: “Quanto tempo vai durar o tratamento?” (tomou apenas uma vez; até melhorar/curar; não vai mais tomar; sempre que voltarem os sintomas; para sempre).

A fim de garantir melhor qualidade da informação, foi solicitado ao indivíduo que mostrasse a(s) caixa(s) ou a(s) receita(s) do(s) medicamento(s).

Foram considerados BZD os medicamentos classificados segundo o *Anatomical Therapeutic Chemical Index* (ATC Index), desenvolvido pelo *World Health Organization Collaboration Center for Drugs Statistic Methodology* ^11^ , sob os códigos N05BA (derivados BZD ansiolíticos) e N05CD (derivados BZD hipnóticos) comercializados no Brasil, além do clonazepam, classificado como anticonvulsivante sob código N03AE01. Foram coletadas ainda informações quanto à utilização de fármacos relacionados aos BZD comercializados no Brasil (zolpidem e zopiclone), sob o código N05CF ^11^ , embora esses medicamentos não tenham sido incluídos no desfecho principal por não pertencerem à classe dos BZD.

As variáveis independentes englobaram fatores sociodemográficos, comportamentais e relacionados à saúde:

a.Sociodemográficos: região (Norte; Nordeste; Centro-Oeste; Sudeste; Sul); sexo (masculino; feminino); idade (anos: 60 a 69; 70 a 79; 80 ou mais); cor da pele autorreferida (branca; preta; parda; amarela; indígena); situação conjugal (sem companheiro[a]; com companheiro[a]); escolaridade (anos de estudo: 0 a 4; 5 a 8; 9 a 11; 12 ou mais); classificação econômica (A-B [mais ricos]; C; D; E [mais pobres]), de acordo com a classificação da Associação Brasileira de Empresas de Pesquisa (ABEP) ^12^ ;

b.Comportamentais: tabagismo (tabagista atual; ex-tabagista; não tabagista); consumo abusivo de álcool no último mês (sim; não), definido como abusivo o consumo de quatro ou mais doses de bebida alcoólica (mulher) ou cinco ou mais doses (homem), em uma única ocasião, pelo menos uma vez no último mês, sendo uma dose de bebida alcoólica equivalente a uma lata de cerveja, uma taça de vinho ou uma dose de cachaça, whisky ou qualquer outra bebida alcoólica destilada;

c.Relacionados à saúde: plano de saúde (sim; não); depressão (sim; não), avaliada por meio da pergunta: “Algum médico já lhe disse que o(a) Sr.(a) tem depressão?”; multimorbidade (sim; não), considerando-se como multimorbidade a presença de duas doenças crônicas ou mais. Foram incluídas as doenças: pressão alta, diabetes, doença cardíaca, colesterol alto, isquemia ou derrame cerebral, asma, doença pulmonar obstrutiva crônica (enfisema ou bronquite crônica) e doença reumatológica. A presença ou não dessas doenças foi avaliada por meio da pergunta: “Algum médico já lhe disse que o(a) Sr.(a) tem [doença]?”; visita à emergência ou internação hospitalar nos últimos 12 meses (sim; não); polifarmácia (sim; não), considerando-se como polifarmácia a utilização atual de cinco medicamentos ou mais (excluindo os BZD), incluindo homeopatia, fórmulas feitas em farmácia de manipulação, florais, vitaminas e fitoterápicos; e autopercepção de saúde (muito boa; boa; regular; ruim/muito ruim).

Foi realizada análise descritiva das variáveis independentes para caracterização da amostra, sendo calculadas as proporções e respectivos intervalos de confiança de 95%. Foi calculada a prevalência de utilização de BZD nos 15 dias anteriores à coleta dos dados, com seu respectivo intervalo de confiança de 95%, geral e conforme as variáveis independentes. Com o uso dos pesos pós-estratificação, perde-se a relação direta entre o número de observações e os percentuais, assim, o número de observações amostrais (n) foi mantido somente nos títulos das tabelas. Foi empregada regressão de Poisson com variância robusta, para obtenção das razões de prevalência, brutas e ajustadas. Na análise ajustada, seguiu-se modelo hierárquico em cinco níveis, construído com base na literatura. O primeiro nível incluiu as variáveis região do país, sexo, idade e cor da pele; no segundo nível constou as variáveis ABEP, escolaridade e situação conjugal; o terceiro nível, por sua vez, integrou as variáveis plano de saúde, tabagismo, depressão e multimorbidade; no quarto nível, a variável visita à emergência ou internação hospitalar nos últimos 12 meses; e o quinto e último nível abarca as variáveis polifarmácia, consumo abusivo de álcool no último mês e autopercepção de saúde. As variáveis foram ajustadas para as do mesmo nível e do(s) nível(is) superior(es); foi utilizado o modo de seleção para trás, sendo mantidas no modelo final aquelas com valor de p < 0,20. Considerou-se nível de significância de 5%. Os dados foram analisados com o programa Stata versão 15.1 (StataCorp, College Station, Texas, USA).

A PNAUM foi aprovada pela Comissão Nacional de Ética em Pesquisa (Protocolo 18947013.6.0000.0008) e pelo Comitê de Ética em Pesquisa da Universidade Federal do Rio Grande do Sul (Protocolo 19997). As entrevistas foram realizadas após leitura do termo de consentimento livre e esclarecido e assinatura do entrevistado ou seu responsável legal.

## RESULTADOS

Foram incluídos neste estudo 9.019 indivíduos que, após o ajuste por região, sexo e idade, passaram a representar os aproximadamente 23 milhões de idosos residentes na zona urbana do país.

Predominaram mulheres (57,7%), indivíduos com idade entre 60 e 69 anos (52,3%), de cor de pele branca (52,8%), pertencentes à classe econômica C (54,7%), que viviam com companheiro (55,8%) e residiam na região Sudeste (52,5%). Do total, 9,5% e 41,5% apresentavam depressão e multimorbidade, respectivamente, e 21,8% eram usuários de polifarmácia. Cerca de 4% relatou consumo abusivo de álcool no último mês, com ampla diferença entre os sexos (mulheres: 1%; IC95% 0,7–1,5 versus homens: 8,3%; IC95% 7,0–9,8). Quase metade da amostra apresentava autopercepção de saúde boa ( Tabela 1 ).

**Tabela 1:** Descrição da amostra, segundo variáveis sociodemográficas, comportamentais e relacionadas à saúde. PNAUM, 2014 (n = 9.019).

Variáveis	% ^a^	IC95%
Região		
Norte	4,8	3,8–6,1
Nordeste	21,2	17,2–25,8
Centro-Oeste	6,8	5,4–8,5
Sudeste	52,5	46,5–58,4
Sul	14,7	11,8–18,1
Sexo		
Masculino	42,2	40,7–43,8
Feminino	57,7	56,2–59,3
Idade		
60–69 anos	52,3	50,7–53,9
70–79 anos	32,4	31,0–33,9
80 anos ou mais	15,2	14,1–16,4
Cor da pele		
Branca	52,8	49,8–55,8
Preta	9,7	8,5–11,0
Parda	36,0	33,4–38,7
Amarela	1,2	0,9–1,6
Indígena	0,3	0,2–0,4
ABEP		
A–B	21,6	19,5–23,9
C	54,7	52,7–56,6
D	19,0	17,1–21,1
E	4,6	4,0–5,5
Escolaridade		
0–4 anos	38,9	36,8–40,9
5–8 anos	20,1	18,8–21,4
9–11 anos	30,1	28,5–31,7
12 anos ou mais	10,9	9,7–12,2
Situação conjugal		
Sem companheiro	44,2	42,3–46,1
Com companheiro	55,8	53,9–57,6
Plano de saúde		
Não	73,0	70,1–75,6
Sim	27,0	24,3–29,9
Tabagismo		
Nunca fumou	62,5	60,3–64,7
Ex-tabagista	26,9	25,1–28,8
Tabagista atual	10,6	9,6–11,6
Depressão		
Não	90,5	89,4–91,4
Sim	9,5	8,6–10,6
Multimorbidade		
Não	58,5	56,5–60,5
Sim	41,5	39,5–43,5
Visita à emergência ou internação hospitalar nos últimos 12 meses		
Não	78,7	77,2–80,2
Sim	21,3	19,8–22,8
Polifarmácia		
Não	78,2	76,5–79,8
Sim	21,8	20,2–23,5
Consumo abusivo de álcool do último mês		
Não	95,9	95,2–96,5
Sim	4,1	3,5–4,8
Autopercepção de saúde		
Muito boa	8,0	7,2–8,9
Boa	48,9	47,0–50,7
Regular	36,0	34,4–37,5
Ruim/Muito ruim	7,1	6,4–7,9

^a^ Percentuais ajustados por pesos amostrais e por pós-estratificação, segundo idade e sexo.

A prevalência de utilização de BZD em idosos nos 15 dias anteriores à coleta dos dados da pesquisa foi de 9,3% (IC95% 8,3–10,4). Considerando o total de usuários de BZD, 59,3% (IC95% 54,0–64,4%) referiram uso eventual; ainda assim, desses, 36,8% (IC95% 30,8–43,1) responderam que o tratamento iria durar “para sempre” e 27,2% (IC95% 21,9–33,2) que iriam tomar o medicamento “sempre que voltassem os sintomas”. Somente 0,7% (IC95% 0,2–2,4) referiu que não iria mais tomar o medicamento.

Considerando o total de BZD, os que apresentaram maior proporção de utilização foram clonazepam (41,3%; IC95% 36,7–46,0), diazepam (22,2%; IC95% 18,2–26,9), bromazepam (14,5%; IC95% 11,5–18,2) e alprazolam (9,6%; IC95% 7,3–12,5) ( Figura 1 ). Esse padrão manteve-se semelhante em todas as regiões do país. Em relação aos fármacos relacionados aos BZD, a prevalência de utilização de zolpidem em idosos foi de 0,1% (IC95% 0,06–0,2).

**Figura 1:** Proporção de benzodiazepínicos utilizados por idosos brasileiros nos 15 dias anteriores à coleta dos dados da pesquisa. PNAUM, 2014 (n = 699).

A Figura 2 demonstra a prevalência de utilização de BZD em idosos por região do país, estratificada por sexo. Percebe-se que a prevalência de utilização de BZD é consistentemente maior em mulheres, embora com importantes diferenças regionais: enquanto na Região Norte a diferença entre os sexos é pequena, não alcançando significância estatística, nas regiões Sul e Centro-Oeste a variação é ampla, atingindo prevalências aproximadamente duas e três vezes maior em mulheres do que em homens, respectivamente.

**Figura 2:** Utilização de benzodiazepínicos em idosos brasileiros nos 15 dias anteriores à coleta dos dados da pesquisa, segundo região do país e sexo. PNAUM, 2014 (n = 9.019).

As maiores prevalências de utilização de BZD foram encontradas nas seguintes categorias: sexo feminino, regiões Sul e Sudeste, 70 anos de idade ou mais, cor da pele branca, viver sem companheiro, possuir plano de saúde, ter depressão ou multimorbidade, visita à emergência ou internação hospitalar nos últimos 12 meses, polifarmácia, não relatar consumo abusivo de álcool no último mês e autopercepção de saúde ruim ou muito ruim ( Tabela 2 ).

**Tabela 2:** Prevalência e razões de prevalência brutas para utilização de benzodiazepínicos em idosos brasileiros nos 15 dias anteriores à coleta dos dados da pesquisa, segundo variáveis sociodemográficas, comportamentais e relacionadas à saúde. PNAUM, 2014 (n = 9.019).

Variável	Prevalência (%) ^a^	IC95%	RP bruta	IC95%
Região				p < 0,001 ^b^
Norte	1,8	1,3–2,6	0,18	0,12–0,26
Nordeste	8,2	7,1–9,7	0,81	0,66–0,99
Centro-Oeste	6,1	4,8–7,6	0,60	0,46–0,77
Sudeste	10,2	8,5–12,2	1,00	
Sul	11,5	9,6–13,7	1,13	0,92–1,38
Sexo				p < 0,001 ^b^
Masculino	6,0	5,0–7,1	1,00	
Feminino	11,8	10,3–13,4	1,97	1,60–2,43
Idade				p = 0,003 ^b^
60–69 anos	7,9	6,8–9,2	1,00	
70–79 anos	11,0	9,3–12,9	1,39	1,13–1,71
80 anos ou mais	10,6	8,3–13,3	1,34	1,04–1,73
Cor da pele ^d^				p = 0,004 ^b^
Branca	11,1	9,8–12,4	1,00	
Preta	7,8	5,4–11,2	0,71	0,50–1,02
Parda	8,0	6,7–9,5	0,72	0,59–0,89
ABEP				p = 0,867 ^b^
A–B	8,6	7,2–9,3	1,00	
C	9,5	8,4–10,8	1,11	0,87–1,40
D	9,4	7,4–12,0	1,09	0,82–1,44
E	8,8	5,7–13,4	1,02	0,62–1,67
Escolaridade				p = 0,185 ^b^
0–4 anos	10,4	8,8–9,1	1,00	
5–8 anos	8,2	6,7–10,2	0,79	0,62–1,01
9–11 anos	8,7	6,9–10,9	0,84	0,66–1,07
12 anos ou mais	8,4	6,3–10,9	0,80	0,59–1,10
Situação conjugal				p < 0,001 ^b^
Sem companheiro	11,5	9,8–13,4	1,00	
Com companheiro	7,9	6,9–9,1	0,69	0,57–0,83
Plano de saúde				p = 0,005 ^b^
Não	8,5	7,5–9.6	1,00	
Sim	11,5	9,4–13,9	1,34	1,09–1,64
Tabagismo				p = 0,450 ^a^
Nunca fumou	9,3	8,0–10,8	1,00	
Ex-tabagista	8,1	6,4–10,1	0,87	0,68–1,11
Tabagista atual	9,8	7,6–12,7	1,05	0,79–1,41
Depressão				p < 0,001 ^b^
Não	6,0	5,3–10,0	1,00	
Sim	39,9	34,7–45,4	6,59	5,57–7,80
Multimorbidade				p < 0,001 ^b^
Não	6,2	5,3–7,3	1,00	
Sim	13,6	11,9–15,6	2,88	2,37–3,50
Visita à emergência ou internação hospitalar nos últimos 12 meses				p < 0,001 ^b^
Não	7,7	6,8–8,8	1,00	
Sim	15,1	12,8–17,8	1,95	1,60–2,38
Polifarmácia				p < 0,001 ^b^
Não	6,8	6,0–7,8	1,00	
Sim	18,2	15,4–21,3	2,65	2,21–3,19
Consumo abusivo de álcool do último mês				p < 0,001 ^b^
Não	9,4	8,3–10,7	1,00	
Sim	2,3	1,0–5,1	0,25	0,11–0,55
Autopercepção de saúde				p < 0,001 ^c^
Muito boa	2,3	1,2–4,2	1,00	
Boa	6,8	5,6–8,2	2,98	1,59–5,66
Regular	11,5	10,0–13,2	5,03	2,67–9,49
Ruim/Muito ruim	23,0	17,9–29,0	10,0	5,16–19,50

^a^ Percentuais ajustados por pesos amostrais e por pós-estratificação, segundo idade e sexo. Foram mantidas no modelo ajustado somente as variáveis com valor de p < 0,20.

^b^ p de heterogeneidade

^c^ p de tendência.

^d^ Considerando o n reduzido, as categorias “amarela” e “indígena” foram transformadas em *missing* para realização de modelo de regressão.

Após análise ajustada, as variáveis que não permaneceram no modelo hierárquico foram classificação econômica, escolaridade, tabagismo e plano de saúde (p > 0,20). A Figura 3 demonstra as razões de prevalência ajustadas das variáveis mantidas no modelo hierárquico, dentre elas, somente cor da pele e situação conjugal não foram significativamente associadas à utilização de BZD na análise ajustada. A utilização de BZD foi cinco vezes maior em indivíduos com depressão, e aumentou conforme a piora da autopercepção de saúde. Indivíduos que relataram consumo abusivo de álcool apresentaram redução em 58% no risco de utilizarem BZD.

**Figura 3:** Razões de prevalência ajustadas para utilização de benzodiazepínicos em idosos brasileiros nos 15 dias anteriores à coleta dos dados da pesquisa. PNAUM, 2014 (n = 9.019).

^a^ Considerando o n reduzido, as categorias “amarela” e “indígena” foram transformadas em missing para realização de modelo de regressão.

## DISCUSSÃO

Este é o primeiro estudo populacional com representatividade das cinco regiões brasileiras que avaliou a prevalência de utilização de BZD em idosos. A prevalência encontrada foi de 9,3%, superior à encontrada na Arábia Saudita (4%) ^13^ e inferior à relatada na França (31%) ^14^ , Finlândia (31%) ^15^ e Taiwan (43%) ^16^ . Em termos absolutos, entretanto, corresponde a um total de mais de dois milhões de idosos no país, representando um grande impacto em termos de saúde pública.

Os fármacos mais frequentemente utilizados foram clonazepam e diazepam, medicamentos entre os BZD que são fornecidos gratuitamente pelo Sistema Único de Saúde (SUS) ^17^ . A maioria dos estudos brasileiros encontrou padrão semelhante ^18^ , somente uma pesquisa encontrou maior consumo de alprazolam e bromazepam ^2^ , observado na maioria dos países de alta renda onde tende-se a optar por medicamentos mais novos, com meia vida curta. Este estudo, porém, utilizou apenas informações oriundas de farmácias particulares ^2^ .

Após análise ajustada, apresentaram maior prevalência de uso as mulheres, os indivíduos com idade entre 70 e 79 anos, os que apresentavam depressão ou multimorbidade, os que relataram ao menos uma visita à emergência ou internação hospitalar nos últimos 12 meses, os que utilizavam polifarmácia e os que relataram autopercepção de saúde ruim ou muito ruim. Por outro lado, apresentaram menor prevalência de utilização os residentes na Região Norte e os indivíduos que relataram consumo abusivo de álcool no último mês.

O presente estudo apresenta algumas desvantagens, condizentes com o crescente aumento de perdas e recusas observadas nos inquéritos populacionais ^10^ . Dados de caracterização da amostra já publicados mostram taxas de resposta do domicílio de 51,7% e 51,5% para homens e mulheres, respectivamente, proporção subestimada, pois incluiu os domicílios vagos no cálculo. Foi encontrada ainda correlação negativa fraca porém estatisticamente significativa entre a renda média do setor censitário e a taxa de resposta em todas as regiões do país ^10^ . Outros estudos que avaliaram idosos, entretanto, não encontraram associação estatisticamente significativa entre nível socioeconômico e utilização de BZD ^3^ , reduzindo a possibilidade de ocorrência de viés de seleção. As taxas de resposta dos moradores foram semelhantes entre homens e mulheres (90,1% e 93,7%, respectivamente) ^10^ .

Em relação ao sexo do entrevistado, mulheres apresentaram probabilidade cerca de duas vezes maior do que homens de utilizarem BZD, achado consistente na literatura ^19^ . Algumas questões são pautadas para explicar essa diferença: em primeiro lugar, estudos mostram que mulheres apresentam mais transtornos depressivos e ansiosos, enquanto homens apresentam com maior frequência transtornos aditivos e externalizantes ^3^ . Uma vez que os BZD são utilizados especialmente no tratamento dos primeiros, sua utilização pode ser um *proxy* para as diferenças de gênero na prevalência de transtornos mentais ^20^ , fortemente impactadas pelo grau de desigualdade de gênero dentro de uma sociedade patriarcal. Outra questão relevante é que as mulheres buscam mais os serviços de saúde para tratamento de problemas de saúde mental ^3 , 20^ . No contexto dos indivíduos idosos, esse achado tem particular relevância clínica, considerando o risco aumentado de quedas com uso de BZD e o risco elevado de fraturas em mulheres idosas, especialmente fraturas de quadril, que ocorrem com elevada morbimortalidade ^21^ .

Foram detectadas amplas diferenças regionais, sendo a prevalência de utilização de BZD na Região Norte (1,8%) muito inferior à encontrada nas demais regiões. Estudos realizados no Brasil que avaliaram a utilização de BZD na população geral encontraram padrão semelhante ^2^ , com aumento do consumo nas cidades de maior densidade demográfica e maior percentual de médicos ^2^ . Fatores como trânsito caótico, sensação de insegurança, ambiente competitivo, grande apelo consumista e baixa coesão social compõe o estilo de vida das grandes cidades atuais, comprometem o bem-estar de seus habitantes e podem contribuir para a maior utilização de BZD observada nesses locais ^2^ . É importante considerar também que a venda dos BZD é controlada no Brasil desde 1998 ^1^ , de modo que o acesso à prescrição médica constitui um determinante para sua utilização. Assim, é provável que esse acesso seja dificultado nas regiões que contam com menor número de médicos por habitante: enquanto o país tem razão média de 2,27 médicos por mil habitantes, a Região Norte tem taxa de 1,30, 43% menor que a razão média nacional ^22^ .

O diagnóstico prévio de depressão foi a variável independente mais importante para predizer a utilização de BZD em idosos. Indivíduos com história de depressão apresentaram prevalência de utilização de 39,9% e, mesmo após análise ajustada, apresentaram risco cinco vezes maior de utilizarem BZD em relação aos indivíduos sem diagnóstico de depressão. Achados semelhantes foram encontrados em outros estudos que avaliaram a utilização de BZD na população geral e em idosos ^23^ .

Os medicamentos antidepressivos (AD) constituem tratamento de escolha para depressão e são eficazes também para o tratamento de sintomas ansiosos coexistentes. No entanto, os efeitos benéficos dos AD geralmente só são obtidos após várias semanas, período em que os BZD costumam ser prescritos visando um alívio mais imediato dos sintomas ^24^ . O benefício do tratamento combinado, entretanto, não se sustenta por mais de quatro semanas ^25^ . Ainda assim, a prevalência de utilização de BZD por tempo prolongado em idosos é elevada ^24^ . Estudos qualitativos apontam como possível causa, a renovação de prescrições iniciadas por outros médicos, buscando atender às solicitações dos pacientes e evitar prejuízo na relação médico-paciente ^26^ . É importante ressaltar que a dependência aos BZD ocorre dentro de poucas semanas de uso regular e está associada à síndrome de abstinência, caracterizada pela ocorrência de distúrbios do sono, ansiedade e agorafobia após a interrupção do uso, especialmente quando realizada abruptamente; a intensidade dos sintomas pode explicar as recaídas após tentativas de interrupção, resultando na perpetuação do uso ^27^ .

Multimorbidade e polifarmácia apresentaram aumento de 1,4 e 1,3 vezes, respectivamente, na probabilidade de utilizar BZD, achado que reflete maior carga de doença e maior risco de transtornos de ansiedade ^19^ . Ambas as condições são mais prevalentes em idosos e encontram-se associadas a maior risco de iatrogenia, piora funcional, perda da independência e autonomia ^8^ . Usuários de um maior número de medicamentos apresentam ainda risco elevado de interações medicamentosas e eventos adversos como confusão, agitação e delirium. Esses sintomas podem ser confundidos com ansiedade e levar à prescrição de BZD, agravando o quadro ^19^ .

Indivíduos que relataram consumo abusivo de álcool no último mês apresentaram redução na probabilidade de utilização de BZD (RP = 0,42; IC95% 0,19–0,94). A maioria dos estudos que avaliaram consumo de álcool no último mês encontram achados semelhantes ^14 , 18 , 19^ . Sabe-se que o álcool e os BZD compartilham mecanismos de ação comuns ^3^ e potencialmente produzem efeitos comparáveis na ansiedade e no sono basais, levando os indivíduos a sentirem a necessidade de um ou outro, mas não de ambos ^19^ . Essa escolha parece ser influenciada, ao menos em parte, pelo sexo do indivíduo, de modo que o presente estudo encontrou maior prevalência de utilização de BZD nas mulheres e de álcool nos homens. É possível ainda que essa diferença resulte de um menor número de prescrições de BZD entre usuários de álcool, uma vez que o efeito das duas substâncias como depressoras do centro respiratório é sinérgico, podendo resultar em intoxicações graves e potencialmente fatais ^18^ .

Somente um estudo encontrou associação oposta, com abuso de álcool determinando maior utilização de BZD (OR 3,1–IC95% 1,7–5,7) ^3^ . Esse estudo, porém, incluiu a população geral e utilizou um período recordatório de 12 meses, tanto para abuso de álcool quanto para utilização de BZD. Em análise de mediação, os autores notaram que o efeito do álcool sobre utilização de BZD foi tanto direto quanto indireto, passando por sintomas depressivos, inatividade física e distúrbios do sono. Pauta-se ainda que, por apresentarem mecanismos de ação comuns, álcool e BZD poderiam levar à tolerância cruzada. Assim, indivíduos dependentes de BZD podem optar por usar álcool durante os períodos de privação, da mesma forma que a classe dos BZD faz parte do tratamento para síndrome de abstinência do álcool ^3^ . Esse fenômeno poderia justificar também os achados encontrados neste estudo, que avaliou o uso de álcool como determinante mais proximal no modelo hierárquico. Assim, é necessária uma abordagem integrada para o tratamento de ambas as condições.

Visita à emergência ou internação hospitalar nos últimos 12 meses também foi associada a uma maior prescrição de BZD para idosos, com aumento do risco em 1,4 vezes. Estudos recentes demostraram elevada prevalência de idosos recebendo BZD durante a hospitalização, o que é preocupante, visto que novas prescrições de BZD fornecidas a pacientes idosos na alta hospitalar podem levar ao uso crônico do medicamento ^28^ .

Por isso, é importante ressaltar que a prevalência de utilização de BZD sem receita encontrada na literatura varia de 3,3% a 8,4% ^2^ , demonstrando que esforços que visam aumentar a fiscalização sobre a venda do medicamento não são suficientes para resolver o problema, uma vez que a maioria dos usuários obtém o fármaco por meio de prescrição médica. Assim, o uso racional de BZD parte da valorização da educação médica continuada e do estímulo às parcerias multiprofissionais ^2 , 9^ , pautadas no atendimento integral ao paciente idoso, com o objetivo de reduzir os eventos adversos evitáveis, e maximizar a independência e autonomia desses indivíduos ^8^ .

A relevância do tema requer investigação adicional, especialmente no contexto de pós-pandemia de covid-19 ^29^ . Evidências emergentes demonstram impactos dramáticos na saúde mental dos indivíduos, com aumento da ansiedade e do isolamento social devido às políticas de distanciamento físico introduzidas para controle da infecção ^30^ , afetando particularmente os idosos. É provável que a prevalência de utilização de BZD, já elevada, aumente ainda mais nesse contexto, de modo que, uma vez controlada a emergência de saúde pública relacionada à pandemia em si, se fazem necessárias políticas públicas que promovam o uso racional dessa classe de medicamentos.

Apesar das potenciais limitações inerentes a um estudo transversal, especialmente em relação à inferência causal, o presente estudo trouxe dados consistentes e com representatividade nacional, até o momento inexplorados na literatura. A despeito das recomendações contrárias ao uso ^6^ , os resultados revelaram elevada prevalência de utilização de BZD em indivíduos idosos, particularmente naqueles que apresentam quadro de depressão, além de amplas diferenças em relação à região do país e ao sexo do indivíduo.
